# Fight, Flight, or Vote Right? A Systematic Review of Threat Sensitivity in Political Conservatism

**DOI:** 10.3390/brainsci15111191

**Published:** 2025-11-04

**Authors:** Tien Dong, Chiara Lucifora, Simona Massimino, Francesca Ferraioli, Alessandra Falzone, Francesco Tomaiuolo, Giovanni Travaglino, Carmelo Mario Vicario

**Affiliations:** 1 Dipartimento di Scienze Cognitive, Psicologiche, Pedagogiche e Degli Studi Culturali, Università di Messina, 98122 Messina, Italy; tien.vy@studenti.unime.it (T.D.); simona.massimino@unime.it (S.M.); alessandra.falzone@unime.it (A.F.); cvicario@unime.it (C.M.V.); 2 Dipartimento di Filosofia e Comunicazione, Università di Bologna, 40126 Bologna, Italy; 3 Department of Clinical and Experimental Medicine, University of Messina, 98122 Messina, Italy; francesco.tomaiuolo@unime.it; 4 Department of Law and Criminology, Royal Holloway, University of London, London TW20 0EX, UK; giovanni.travaglino@rhul.ac.uk

**Keywords:** political orientation, political conservatism, threat sensitivity, skin conductance level, neurophysiology, emotions, behavior, methodological variations

## Abstract

Background: Within the framework of social cognition, conservatism can be conceptualized as a strategy for addressing fundamental psychological needs. Therefore, it is hypothesized that individuals with conservative orientations exhibit stronger reactions to perceived threats compared to their less conservative counterparts. Aim: To perform an exploratory scoping systematic review of existing literature examining behavioral, physiological, neurophysiological, and emotional responses associated with the relationship between conservatism and threat perception. Method: Following PRISMA guidelines, a systematic search was conducted using PubMed and Google Scholar primary databases, resulting in the inclusion of 19 relevant articles. Results: Approximately three-fifths (11 of 19 studies; 57.9%) provided empirical support for the hypothesis that conservatism is positively associated with threat sensitivity. These findings reveal a complex and nuanced relationship between conservatism and threat perception, with recent evidence—including large-scale longitudinal data and experimental manipulations of COVID-19–related threats—indicating weak or context-dependent associations. The overall pattern highlights substantial heterogeneity across methodological approaches, with mixed results particularly among physiological and priming studies. Conclusions: While the majority of evidence supports a relationship between political conservatism and threat sensitivity, the magnitude of this association appears modest, emphasizing the importance of considering moderating variables such as cultural context, the type of threat, and methodological variations in measurement in future research.

## 1. Introduction

The fight-or-flight response, a core component of fear processing, mobilizes the body’s physiological systems to react rapidly to perceived threats, shaping how individuals detect, interpret, and respond to danger [1]. Heightened sensitivity to such stimuli can influence cognitive and affective dispositions, promoting attitudes oriented towards protection, order, and control—features frequently associated with conservative political ideology [2,3,4].

More recently, Neill [5], as cited in Goldman [6], differentiated liberalism and conservatism through the central concept of hierarchy. According to Neill, conservatives tend to regard inherent differences in ability—expressed through civic engagement, market participation, and other voluntary activities—as legitimate grounds for existing social and political inequalities. This acceptance of hierarchical view of inequality, which is further captured in various measures of conservatism, such as Sidanius and Ekehammar’s (1979) [7] Political–Economic Conservatism scale and Pratto et al.’s (1994) [8] Social Dominance Orientation scale, can also be linked to fear sensitivity because individuals with higher threat sensitivity are more likely to favor stable, ordered, and predictable systems—like those offered by hierarchical structures. Crucially, the preference for hierarchical social structures can be understood in light of threat sensitivity: individuals with higher sensitivity to threat often seek environments that are stable, ordered, and predictable. Hierarchical systems provide precisely this kind of structure, reducing perceived uncertainty and reinforcing social order—features that make them particularly attractive to those with a conservative political orientation [2].

### 1.1. Theoretical Perspectives on Conservatism and Threat Sensitivity

Several theoretical models have been developed to explain the link between conservatism and various social, cognitive, and motivational factors, although they emphasize different underlying aspects.

Adorno et al. [9] proposed a theory of authoritarianism, suggesting that experiences of parental threat or punishment led individuals to idealize authority figures while directing blame and hostility towards outgroups—reflecting a broader drive for predictability and control in one’s environments. In contrast, Wilson [10] argued against equating authoritarianism with conservatism. Instead, he posited that conservatism represents a broader construct, of which authoritarianism is merely one possible expression.

In 1981, Altemeyer [11] introduced a model of Right-Wing Authoritarianism (RWA), offering both a reconceptualization of authoritarianism and a reliable method for its empirical measurement. Notably, in a 1998 study examining the attitudes of legislators, Altemeyer found that individuals with high RWA scores were more likely to endorse core elements associated with conservatism—such as prejudice, restrictions on freedom of speech and the press, opposition to abortion and gun control legislation, and support for capital punishment. Based on these findings, he argued that extreme conservatism, when coupled with authoritarian dispositions, may foster hostility toward out-groups and a submissive orientation toward established authorities. Wilson [12] proposed a dynamic theory of conservatism, centering on the idea that the core driver of conservative behavior is a “generalized susceptibility to experiencing threat or anxiety in the face of uncertainty.” He further differentiated uncertainty into two distinct forms: stimulus uncertainty, which pertains to unpredictability in the external physical and social environment, and response uncertainty, which involves ambiguity within the individual’s own cognitive or emotional processes. According to Wilson, it is a specific type of uncertainty, perceived as threatening, that gives rise to conservative attitudes. These attitudes function defensively, aiming to simplify, control, and secure both the external world and the internal psychological landscape in response to perceived threats.

According to Regulatory Focus Theory [13,14], individuals’ political orientations may be shaped by their underlying motivational focus on two distinct classes of goals: promotion goals, which emphasize advancement and growth, and prevention goals, which prioritize safety and security. These orientations correspond to preferences for change and stability, respectively—where a promotion focus aligns with more progressive or left-leaning attitudes, and a prevention focus aligns with more conservative or right-leaning attitudes. When political conservatism is driven by a need for security and the avoidance of threat or change, individuals are more likely to adopt conservative positions under conditions that activate a prevention-oriented regulatory focus.

Terror management theory (TMT [15,16,17]) posits that cultures and worldviews function as buffers against the anxiety—and existential terror—that arise from the awareness of mortality. In relation to conservatism, research has shown that when individuals are reminded of their mortality, they tend to display more conservative behaviors, such as increased hostility toward outgroups and harsher judgments of those who challenge their deeply held beliefs [16,17]. This supports the idea that, according to TMT, social intolerance may stem from worldview-enhancing cognitions that help manage existential anxiety, suggesting that conservatism is, at least in part, a motivated form of social cognition. However, it is important to note that recent meta-analytic and replication studies have raised concerns about the robustness of TMT findings, with some key effects failing to replicate consistently (e.g., [18]). These findings call for a more cautious interpretation of the theory’s explanatory power and underscore the need for further empirical scrutiny.

System Justification Theory (SJT) posits that individuals are motivated to engage in cognitive and ideological processes that rationalize and legitimize the existing social system, thereby reinforcing the status quo and perpetuating inequality. This occurs even when such behaviors appear to contradict other important needs, such as maintaining group standing (e.g., [19,20,21]). A potential hypothesis derived from SJT [2] is that individuals are motivated to protect and defend the current social system against perceived threats in order to maintain its stability and legitimacy. Social Dominance Theory (SDT, [8,22,23,24,25]) argues that human societies create ideological belief systems to minimize intergroup conflict by rationalizing the dominance of certain groups over others. This aligns with the idea that conservatives are more accepting of social inequality. SDT links threat sensitivity to conservatism by suggesting that individuals may become more supportive of social hierarchies and more resistant to change when they perceive threats to their own status or to the broader social structure.

Motivated Social Cognition Theory (MSCT) [2] offers a framework that integrates epistemic (e.g., uncertainty avoidance, needs for order and closure), existential (e.g., self-esteem, terror management, fear, and threat), and ideological motives (e.g., self-interest, group dominance, and system justification). This theory highlights the central role of threat, fear, and uncertainty in shaping the core tenets of conservatism: resistance to change and the justification of inequality. Jost and colleagues argued that in the face of threat and uncertainty, individuals may embrace specific conservative ideologies based on these underlying motives. They contend that conservatism fundamentally appeals to individuals who are dispositionally or situationally prone to experiencing fear or aversion to uncertainty.

In summary, the relevance of threat as a key factor in explaining conservatism is illuminated by various theoretical models that link psychological responses to threat with conservative attitudes. Theories such as Adorno et al. [9] and Wilson [12] suggest that experiences of threat foster a susceptibility to anxiety, driving individuals to seek predictability and control, which often manifests as conservative behavior. RFT posits that a strong prevention-oriented motivation further fuels conservative attitudes rooted in the need for safety and security. Moreover, TMT demonstrates how thoughts of mortality can lead to increased conservative behaviors and social intolerance as individuals attempt to mitigate existential anxiety. SJT and SDT reinforce these arguments, positing that perceived threats prompt individuals to defend the status quo and support social hierarchies. Collectively, these frameworks underscore that heightened sensitivity to threat is a significant motivator for the adoption and maintenance of conservative beliefs, highlighting conservatism as a defensive response to perceived dangers in people’s environments.

### 1.2. Psychological and Neurophysiological Mechanisms of Threat Response

Reiss et al. [26] proposed a model for the taxonomy of psychological threat, discussing three main dimensions: origin, needs, and defense. In terms of origin, threats are believed to manifest within phenomenological worlds, including the physical environment, social realm, inner experience, and spiritual domain. Consequently, threats can be categorized into two groups: existential threats, which pertain to issues such as death, isolation, identity, freedom, and meaning, and non-existential threats, which are situational and serve as a counterpoint to the former. Similarly, Crawford [27] proposes a Compensatory Political Behavior (CPB) Model which separates threats into meaning type that elicits anxiety and physical type that induces fear. However, the model also posits that both liberalism and conservatism will be equally influenced by meaning threats, while physical threats will be more significant in relation to conservatism, especially for social conservatives. In these phenomenological contexts, threats can jeopardize an individual’s relationship with these realms, relating to three essential psychological needs: autonomy, competence, and relatedness. Therefore, individuals must fulfill these needs as a means of self-defense and coping with the threats they experience.

Responses to perceived threats can be immediate. For instance, mortality salience primarily evokes negative emotional responses, including anxiety, fear, and sadness [28]. Poppelaars et al. [29] found that existential threats elicit an increase in self-reported negative emotions, such as feelings of distress, upset, and unhappiness. Neurophysiological studies have shown that threats related to mortality and uncontrollability activate the anterior cingulate cortex [30]. Furthermore, Eippert et al. [31] noted increased amygdala activity in response to threatening events, which is consistent with findings from Schaefer et al. [32] and Ochsner et al. [33]. From a physiological perspective, threat stimuli lead to increased activity in various physiological indicators such as skin conductance, heart rate, and startle reflex [34,35,36,37,38,39,40]. However, the physiological responses to existential and non-existential threats are largely similar [29].

According to Jonas et al. [41], all threats trigger a distal defense mechanism that downregulates the neural processes associated with anxiety stemming from discrepancies in experience. The General Process Model of Threat and Defense (GPM), also proposed by these researchers, analyzes this mechanism across two dimensions: from concrete to abstract and from personal to social. For instance, a concrete personal defense may involve individuals committing tangible rewards. In this context, threats such as mortality salience are likely to increase indulgent consumer choices [42] or lead to heightened materialism [43]. Conversely, social concrete defense is characterized by a desire for social connections following a threat. Maner et al. [44] found that threats related to social exclusion can increase individuals’ interest in interacting with others, even with strangers.

In the realm of abstract defenses, personal responses often focus on unattainable ideals and values. For example, mortality threats may compel individuals to cultivate a more positive self-image [45] or may result in conservatives becoming more biased while liberals exhibit increased tolerance towards differences among others [46].

Regarding abstract social defense, when people face a threat, they often adjust their behavior to support the group’s shared values or identity, helping to protect the group’s image or sense of unity. Research shows that when people are reminded of death, feel uncertain, experience a lack of control, or encounter unexpected events, they tend to show stronger support for their in-group’s beliefs and ideologies [47,48,49]. In summary, Reiss et al. [26] present a comprehensive model for understanding psychological threats, categorizing them based on their origin and impact on essential psychological needs such as autonomy, competence, and relatedness. By identifying and distinguishing between existential and non-existential threats, the model highlights how these threats can disrupt an individual’s relationship with various phenomenological worlds. The immediate emotional and physiological responses to such threats, including increased anxiety and distinct neural activity, underline the profound effects of perceived danger on well-being. Furthermore, Jonas et al. [41] expand on this by proposing the General Process Model of Threat and Defense, which explains how individuals engage in diverse coping strategies—ranging from concrete actions to abstract idealizations—to mitigate the effects of threats. This integration of emotional, physiological, and defense responses provides a nuanced understanding of how individuals navigate the complexities of threat and demonstrates the significant interplay between psychological factors and social dynamics in shaping human behavior in the face of adversity.

Drawing on the MSCT [2], which posits that the core ideology of conservatism is characterized by resistance to change and acceptance of inequality, it is suggested that these attitudes are influenced by situational and positional needs to address perceived threats. This threat sensitivity framework converges with several complementary theories. SJT [2] proposes that individuals are motivated to defend and legitimize existing social hierarchies, a process that may be intensified under conditions of uncertainty and threat. Similarly, TMT [15] argues that existential anxiety in the face of mortality salience fosters stronger adherence to conservative worldviews that provide order, stability, and protection. Finally, SDT [25] emphasizes that preference for group-based hierarchies is partly sustained by perceiving outgroups as threatening, legitimizing inequality as a defensive strategy. Taken together, these frameworks suggest that right-wing ideological orientations may be particularly responsive to fear and threat cues.

Beyond its psychological and sociological implications, the study of threat sensitivity also provides a window into the neurocognitive mechanisms underlying ideological cognition and emotional regulation. Evidence from neuroimaging research [50] highlights the involvement of key neural circuits in threat processing, particularly the amygdala, bed nucleus of the stria terminalis (BNST), and prefrontal regions, which collectively mediate the detection, appraisal, and regulation of perceived threats. These neural systems not only govern basic affective responses but also shape higher-order cognitive evaluations related to social conformity, uncertainty reduction, and risk avoidance—processes that are central to political conservatism.

From this perspective, political ideology can be conceptualized as a motivational and neurocognitive adaptation to perceived threat, integrating biological, emotional, and cognitive dimensions. This view resonates with major theoretical frameworks such as the MSC model, SJT, and TMT, which converge on the idea that ideological orientation may emerge from the interaction between neural threat systems and motivational needs for stability and control.

Accordingly, this systematic review aims to examine the relationship between threat perception and conservatism, focusing on behavioral, physiological, neurophysiological, and emotional responses.

In addition to its theoretical relevance, the topic of threat sensitivity and political orientation has gained renewed importance in light of recent global events. The COVID-19 pandemic represented an unprecedented worldwide threat that reshaped individuals’ perceptions of safety, uncertainty, and social cohesion [51,52,53,54]. This crisis not only heightened existential and health-related anxieties but also amplified ideological polarization and group-based responses to perceived danger. For example, Nowlan and Zane [55] found that heightened perceptions of the COVID-19 threat led conservatives to adopt protective behaviors more similar to liberals. Similarly, Syfers et al. [56] demonstrated that under pandemic-related uncertainty, conservatives reported greater symbolic and realistic threat perceptions, which in turn shaped their engagement in risk-related behaviors. Together, these findings illustrate how large-scale crises can activate the psychological and motivational mechanisms underpinning threat sensitivity, thereby influencing political attitudes and behaviors on a global scale.

Previous works [57] provided evidence that fear and threat responses tend to provide a small-to-moderate political advantage to conservative causes. Our review incorporates these more recent findings, providing a more up-to-date and comprehensive synthesis of the evidence. Critically, our work specifically examines studies that incorporate physiological measures in relation to conservatism. Additionally, the review will conclude with a discussion of potential implications for future research directions in this field.

## 2. Methodology

Our work was conceived as an exploratory scoping systematic review of existing literature examining behavioral, physiological, neurophysiological, and emotional responses associated with the relationship between conservatism and threat perception. This study adheres to the Preferred Reporting Items for Systematic Reviews and Meta-Analyses (PRISMA) guidelines [58]. The identification of relevant papers was conducted from July to October 2025 across the databases of Google Scholar, PubMed, utilizing various keywords, including “political conservatism,” “conservatism,” “political ideology,” “threat,” “threat sensitivity,” and “physiological response,” within the publication years 2008, which refers to the first publication in the field [59] to 2024. Additional records (other sources) were identified only through manual cross-referencing of citations within eligible articles, rather than through separate databases.

**Figure 1:**
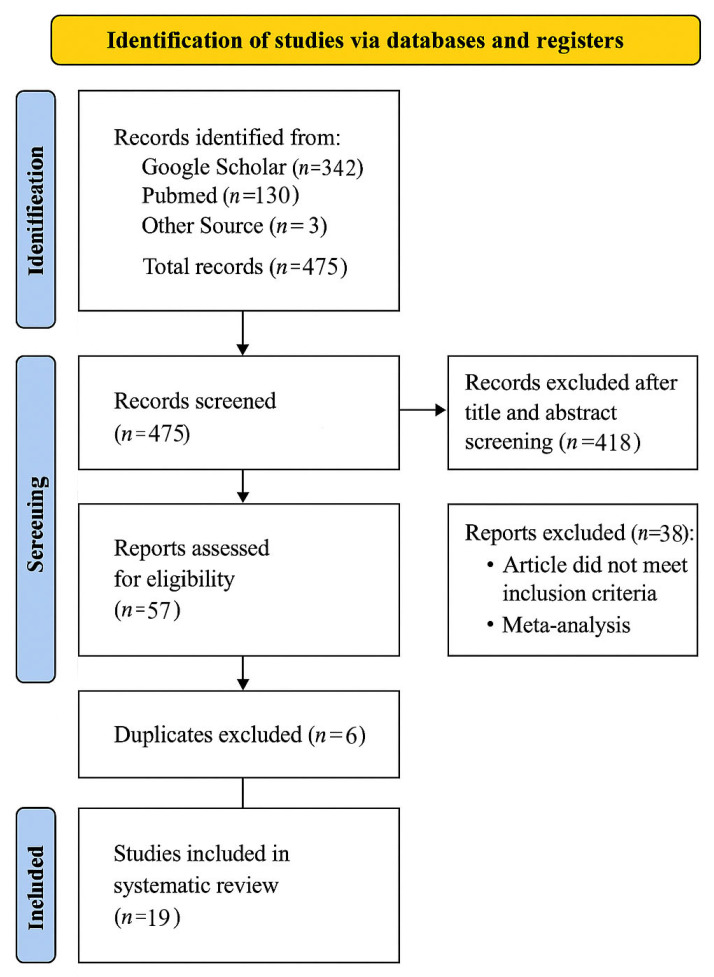
PRISMA flowcharts shows the identification, inclusion, and exclusion of studies in the systematic review.

After duplicate removal, all retrieved records (N = 475) were screened in accordance with PRISMA 2020 guidelines. The initial title and abstract screening was conducted to identify studies explicitly examining the relationship between threat sensitivity and political conservatism. Full-text articles were then assessed based on the following inclusion criteria: (a) publication in English; (b) empirical studies presenting original data (excluding reviews or meta-analyses); (c) direct investigation of threat sensitivity—behavioral, physiological, neurophysiological, or emotional—in relation to political conservatism. Studies were excluded if they (a) focused solely on disgust sensitivity or other affective constructs unrelated to threat; (b) were theoretical or conceptual papers without empirical data. The progressive reduction from 475 initial records to 19 included studies is documented in the PRISMA flow diagram (Figure 1), which details the identification, screening, eligibility, and inclusion phases.

We did not consider “disgust sensitivity” among the keywords because our focus was explicitly on threat sensitivity as a psychological and physiological construct, rather than on related but distinct affective dispositions. While disgust sensitivity has been discussed in the literature as a potential correlate of political orientation (e.g., [4]), it is conceptually narrower and primarily pertains to pathogen avoidance and moral purity domains [60,61,62]. These do not encompass the broader spectrum of threat-related responses—such as fear, anxiety, or vigilance toward physical, social, and symbolic threats—examined in our review. Including “disgust sensitivity” as a search term would have substantially broadened the scope beyond our primary research question and risked introducing studies that address adjacent but theoretically distinct constructs.

No pre-registration was performed. The inclusion criteria for selecting articles required that studies be published in English and investigate the relationship between conservatism and threat in terms of both immediate responses and distal defense mechanisms. In terms of exclusion criteria, reviews and meta-analyses were excluded from consideration, as our aim was focusing on original data instead of synthesis of existing research.

The search process involved combinations of the specified keywords to identify relevant articles. Following this, the articles were screened based on their titles and abstracts, and it was verified whether they could be fully retrieved. The remaining studies were then assessed against the established eligibility criteria for inclusion.

Overall, 475 articles were identified across Google Scholar, PubMed, and other sources; however, after assessment, only 19 articles were selected for review.

The selected papers are organized based on measurement modalities as follows: physiological measurement, self-report, behavioral outcomes, and neuroimages. In the summary section of the results, the articles will be presented in chronological order, ranging from the earliest to the most recent. Detailed information regarding all the studies included in this review, including the risk of bias, can be found in the Supplementary Materials. The flow diagram outlining the study selection process is shown in Figure 1.

Regarding study quality, the risk of bias was assessed using the JBI Critical Appraisal Tools and Cochrane methods. Overall, by using the JBI tools all quantitative studies were assessed with scores higher than 73% although four studies were deemed to have some concerns and two studies to have serious risk of bias due to confounding factors. Among the studies employing trial designs, three non-randomized studies were identified as having a serious risk of bias—two due to confounding factors and one due to participant selection and missing data. One randomized controlled trial was included and presented some concerns related to potential missing outcome data. Full details are provided in Tables S5–S7 in the Supplementary Materials.

## 3. Results

Building on the theoretical framework presented above, this section reports the empirical findings emerging from the reviewed studies, highlighting how different methodological approaches (physiological, behavioral, self-report, and neuroimaging) converge or diverge in supporting the proposed relationship between conservatism and threat sensitivity. All available quantitative information, including the sample size of each included study, the corresponding statistical outcomes, and effect size indicators (when reported in the original sources), is provided in the Supplementary Materials (Summary Tables—Tables S1–S4). Further details regarding study quality, confounding factors, and risk of bias are available in the Supplementary Materials (Tables S5–S7).

### 3.1. Physiological Studies

Oxley et al. [59] were the first to examine the relationship between political attitudes and physiological reactivity. Using the Wilson–Patterson scale, participants’ opinions were assessed across 18 policy issues, including support for military spending, warrantless searches, the death penalty, the Patriot Act, obedience, patriotism, the Iraq War, school prayer, and Biblical truth; and opposition to pacifism, immigration, gun control, foreign aid, compromise, premarital sex, gay marriage, abortion rights, and pornography. Additionally, sociodemographic variables such as age, gender, income, and educational level were controlled for. Physiological responses were measured by change in skin conductance level (SCL) and orbicularis oculi startle blink reflex while participants were exposed to threatening stimulus.

The findings revealed a significant difference in SCL: individuals more supportive of protecting social units exhibited elevated responses, whereas those showed little to no change. This indicates a positive association between physiological reactivity to threat and social conservatism. A similar pattern emerged for the startle blink response, although this difference did not reach statistical significance.

Dodd et al. [63] examined the association between political attitudes and threat response from both physiological and attentional perspective; however, for consistency, only the physiological findings are reported here. In addition to the Wilson–Patterson index, participants provided self-reports of ideological position, partisan affiliation, and opinions on specific social principles. Electrodermal activity (skin conductance level, SCL) was recorded using Ag/AgCl electrodes while participants viewed appetitive and aversive images. Results showed that although electrodermal activity increased in response to both types of images were presented; individuals on the political right exhibited significantly greater reactivity to negative images, whereas those on the political left showed stronger responses to positive ones.

For left-wing participants, the pattern was reversed, with stronger responses to positive images. In a follow-up experiment, the researchers tested reactions to ideologically congruent (appetitive) and incongruent (aversive) political figures and found the same consistent pattern. Taken together, these findings suggest a positive association between right-wing orientation and heightened threat reactivity to negative stimuli.

Building on prior work on physiological responses, Arceneaux et al. [64] examined policymakers in the United States. State legislators and their staff were asked how they would allocate the state budget across six policy areas: four aligned with liberal priorities (education, higher education, healthcare, and assistance to the poor) and two with conservative priorities (public safety and counterterrorism). Electrodermal activity (SCL) was recorded as participants viewed threatening images. Results showed that individuals with higher electrodermal reactivity were more likely to shift resources toward conservative priorities, suggesting that conservatism may function as a defensive mechanism aimed at minimizing threat.

Knoll et al. [65] sought to replicate the findings of Oxley et al. [59] and Dodd et al. [63] using a new dataset and context, but their results contradicted prior evidence. As in earlier studies, they measured SCL responses to threatening stimuli, though they did not include the eye-blink reflex used by Oxley et al. [59] To capture political orientation, participants rated agreement with five public policy statements from the Pew Values Survey, where higher scores reflected socially conservative preferences. Contrary to the original findings, individuals favoring protective policies exhibited a lower average change in SCL compared to those who did not. For the replication of Dodd et al. [63], the authors constructed a broader 12-item scale incorporating ideology, partisan affiliation, and policy preferences. Again, the key findings were not supported: no significant difference emerged in SCL reactivity to aversive images between left- and right-leaning participants. Taken together, these results suggest that the previously reported positive association between conservatism and threat sensitivity was not observed in this replication.

Bakker et al. [66] conducted three direct replications of Oxley et al. [59] in both the USA and the Netherlands, utilizing larger samples and closely following the original protocol. Despite these efforts, they were unable to reproduce the original findings: no significant associations were observed between either social and economic conservatism and SCL responses to threatening or disgust-eliciting stimuli. Some correlations did emerge when additional physiological measures, such as corrugator muscle activity and labial responses, were considered. The researchers also attempted to replicate the findings of Dodd et al. [63] but again found no supporting evidence.

In another replication attempt, Osmundsen et al. [67] conducted a cross-country study comparing the United States and Denmark. Electrodermal activity was recorded as participants viewed images categorized as threatening, disgusting, positive, or neutral. In addition to the Wilson–Patterson items from the original study, the researchers included measures of social conservatism, economic conservatism, and ideological self-placement, with higher scores indicating stronger conservative orientation. Among U.S. participants, greater electrodermal activity was associated with support for conservative policies on the Wilson–Patterson and social conservatism scales, consistent with Oxley et al. [59], although no significant correlations were observed with the economic or ideological scales. In contrast, the Danish sample showed no relationship between conservatism and electrodermal reactivity; if anything, stronger responses to threatening stimuli were (non-significantly) associated with liberal preferences.

Most recently, Arceneaux et al. [68] attempted to replicate the association between conservatism and unconscious threat responses in light of previous failed reproductions. In addition to standard measures of social conservatism, they employed the iatgen R package with liberal- and conservative-related word stimuli to assess implicit ideology. Threat sensitivity was measured through both SCL and self-reported arousal, following the approach of Osmundsen et al. [67]. Results showed that the physiological negativity bias linked to social conservatism in earlier studies (e.g., [59,64]) could not be reproduced. A weak association did emerge between self-reported arousal and social conservatism, but no correlation was found with implicit ideology. Given these inconsistencies, a second study with a larger sample was conducted, yet the association between self-reported arousal and social conservatism was effectively null. Moreover, analyses of economic conservatism revealed a negative, statistically insignificant relationship.

In summary, research on the relationship between political attitudes and physiological responses to threat reveals a complex and contested picture. Early studies by Oxley et al. [59] and Dodd et al. [63] reported a positive association between conservatism and heightened physiological reactivity, suggesting that political orientation might shape threat sensitivity. However, subsequent replication attempts have produced inconsistent findings. Knoll et al. [65] observed lower SCL changes among conservatives favoring protective policies, contradicting the original results. Osmundsen et al. [67] found that electrodermal activity correlated with conservative preferences in a U.S. sample but not in Denmark, underscoring the influence of cultural context. Direct replications by Bakker et al. [66] further challenged the robustness of the original findings, reporting weak and nonsignificant correlations overall. More recently, Arceneaux et al. [68] also failed to reproduce the link, despite testing both explicit and implicit measures of conservatism. Collectively, these studies suggest that the association between threat sensitivity and political orientation is less consistent than once believed. Variability across contexts and populations points to the need for further research into underlying mechanisms and sociocultural influences. This body of work highlights both the challenges of integrating physiological measures with political ideology and the need for new approaches to better understand their interplay.

### 3.2. Self-Reported Studies

Eadeh and Chang [69] conducted three studies examining whether threat increases support for liberal policies. Across domains such as health care, pollution, and finance, they found that threat heightened endorsement of liberal attitudes, showing that threat does not invariably shift ideology to the right but can also bolster left-leaning positions. Similarly, Nowlan and Zane [55] explored responses to disease threats during the COVID-19 pandemic. Conservatives, who initially perceived the virus as having low agency, were less likely to assign blame or anticipate a second wave. However, when agency perceptions increased, conservatives—like liberals—became more inclined to attribute blame and adjust consumption behaviors. These findings suggest that heightened threat perception can motivate conservatives to adopt protective measures, supporting the view that ideological responses to threat are context-dependent and shaped by perceived relevance to one’s own interests.

Brandt et al. [70] investigated how political orientation relates to different types of threat, taking into account political domain and national context. Using 60,378 responses from the World Values Survey across 56 countries, they assessed economic and cultural identification alongside perceived threats such as violence, neighborhood safety, policing, economic insecurity, poverty, and surveillance. The study found significant associations between right-wing orientation and six types of threat, as well as between left-wing orientation and nine types. Notably, these associations varied across countries, with only a few contextual factors—such as government effectiveness, Eastern Bloc history, economic conditions, and ideological constraints—helping to explain cross-national variation in the threat–political orientation link.

Pazhoohi and Kingstone [71] examined the impact of pathogenic threat during COVID-19 on social cognition, focusing on authoritarianism and political conservatism. Data were collected in the United States during the early stage of the pandemic, when perceived threat was low, and one month later, when threat perception had intensified. As reported COVID-19 cases increased, individuals who expressed greater concern about infection also scored higher on right-wing authoritarianism and conservatism, suggesting a positive correlation between conservatism and threat sensitivity. Similarly, Lukaszewicz [72] investigated the relationship between societal threat and political ideology using data from 1013 participants in 2018. Political orientation was measured with Wilson–Patterson items, and conservatism was categorized as social, economic, or securitarian. While liberals initially reported greater threat across both social and asocial domains, further analysis revealed that economic conservatives expressed less fear overall, whereas social conservatives—and especially securitarian conservatives—exhibited heightened perceptions of threat in social contexts.

Building on their earlier cross-national study, Osmundsen et al. [67] supplemented physiological measures with self-reported emotional reactions to threatening images. Unlike the null physiological findings, participants who rated the images as more negative also scored higher on conservatism in both the Danish and American samples, though this effect was significant only for social conservatism. Brown et al. [73] investigated the relationship between political attitudes and disease threat through two studies on environmental pathogen stress. In the first, they analyzed state-level data from 1960 to 2006, finding that conservatism correlated positively with parasite stress, though the effect diminished over time. In the second, age was tested as a moderator, revealing that the conservatism–pathogen stress link was stronger among older individuals, particularly those aged 40 and above.

Syfers et al. [56] conducted two correlational studies examining how political orientation, self-uncertainty, perceived threats (symbolic and realistic), and risky behaviors interacted during COVID-19. The first study found that higher conservatism was positively associated with both symbolic and realistic threats under conditions of self-uncertainty. The second study replicated these correlations and added risky social behaviors as an outcome, showing that self-uncertainty predicted greater engagement in risky behaviors among conservatives. These findings suggest that perceived threats to freedom may drive such behaviors, consistent with the MSC model.

Wang et al. [74] further extended this line of research by experimentally testing the effects of perceived COVID-19 economic threat on political conservatism, xenophobia, and racial bias in two U.S. samples. Across studies, exposure to economic threat (versus control conditions) increased xenophobic attitudes and indirectly heightened conservatism and racial bias through perceived group-status threat. These effects were stronger among participants financially impacted by the pandemic. The study represents the only randomized controlled trial identified in this review and was assessed as having a low risk of bias (93.8%), with minor concerns related to sample representativeness.

Cassario and Brandt [75] analyzed nationally representative samples from the Netherlands and the United States to examine ideological responses to various ecological threats, including immigration, unemployment, violent crime, racial diversity, and the COVID-19 pandemic. In contrast to earlier findings, the authors observed no systematic association between perceived threat and conservatism, reporting mixed results across contexts.

Taken together, these self-reported studies demonstrate a complex and context-dependent relationship between threat perception and political conservatism. Conservatives often display heightened sensitivity to threats ranging from health and economic challenges to social and existential uncertainties, particularly when their security or core values are perceived as being at risk. However, the direction and magnitude of these associations are not uniform. In certain contexts—such as during the COVID-19 pandemic or across large-scale longitudinal samples—threat exposure has not consistently predicted stronger conservative attitudes (e.g., [75]). Overall, the findings suggest that self-reported threat sensitivity among conservatives reflects both a dispositional orientation toward security and stability and a flexible adaptation to situational and cultural factors. Thus, self-report measures capture both the relative stability and contextual variability of threat perception within political ideology.

### 3.3. Behavioral Studies

In the same article by Dodd et al. [63], the authors designed an eye-tracking study to observe participants’ attentional patterns when viewing aversive and appetitive images. In addition to the same procedure used in the physiological study, participants were also asked to self-identify their party affiliation. For the recording of gazing behavior, two measurements were employed: dwell time, which indicates the amount of time spent on each image, and first fixation time, which shows the time elapsed from the onset of the trial until participants moved between each image type. For dwell time measurements, it was found that political right individuals spent more time looking at aversive images while those who identified as left devoted more attention to appetitive ones. Similarly to the other measurement, the political left was quicker to fixate on appetitive images, whereas the opposite was true for the right.

Mills et al. [76] conducted a study to test the relationship between memory for negative pictures and political ideology. In the study, participants completed eight catch trials to measure their memory responses. Findings demonstrate that, in general, negative pictures were more memorable; however, the effect was stronger for conservative ideology. However, between type of negative pictures, memory for animal mistreatment and human subtype were stronger relative to threat-related images. As a result, the correlation between political ideology and negativity bias, or threat, can be extended to memory.

In summary, reviewed studies provide insights into the relationship between political orientation and emotional responses to aversive stimuli. Dodd et al. [63] demonstrated that individuals identifying as politically right tend to focus more on aversive images, while those on the left are more attuned to appetitive images, indicating distinct attentional patterns. Pedersen et al. [50] explored the neural underpinnings of this relationship, identifying increased connectivity between the amygdala and BNST in conservatives under threat conditions. Meanwhile, Osmundsen et al. [67] highlighted the importance of self-reported emotional reactions, finding that perceiving threatening images negatively correlates with conservatism. Finally, Nash and Leota [77] indicated that conservatives exhibit heightened sensitivity to negative stimuli, particularly under economic threat, as evidenced by Electroencephalography (EEG) measures. Collectively, these findings highlight the nuanced ways in which political beliefs influence emotional processing and attentional focus in response to threats.

### 3.4. Neuroimages Studies

From a neural perspective, Pedersen et al. [50] were the first to study the correlation between conservatism and threat sensitivity by examining the connectivity changes between the amygdala and the bed nucleus of the stria terminalis (BNST). In the study, instead of using threatening images, the threat of shock was induced. Before the scan, the participants were presented with the instruction: “You will be under threat of shock during this scan, meaning that you may receive the electrical stimulus at any time during this scan. You may receive multiple electrical stimulations during this scan”, and they were asked to perform a task during the scanning process. Unlike the physiological evidence in which social conservatism correlates with threat sensitivity, the researchers found that under the threat of shock, specifically, economic conservatism predicted greater connectivity between a cluster in the left amygdala and the BNST, while the connectivity became weakened under the safe conditions. In the study, social conservatism was also measured but was excluded during the analysis process due to the lack of variation in the samples. Nash and Leota [77] conducted two EEG studies to investigate whether political orientation functions as a psychological defense mechanism or reflects heightened dispositional sensitivity to negative stimuli. In the first study, participants were randomly assigned to either an Economic Threat condition or the Control condition. After that, they performed an auditory startle task while the P3 component of the white noise was being indexed using EEG recordings. Finally, they provided self-reports of their affective responses to the economic threat manipulation. Results showed that under economic threat, conservative displayed significantly greater P3 mean amplitude and a larger difference wave compared to the control condition. This effect did not occur for individuals with a liberal orientation, suggesting that conservatives become more sensitive to negative stimuli when facing economic threats. The second study replicated this pattern with a larger sample and extended the analysis using source localization. Here, conservatives again showed heightened P3 responses under threat, accompanied by significantly increased activation in the dorsomedial prefrontal cortex and dorsal anterior cingulate cortex relative to controls. Collectively, neuroimaging evidence suggests that conservative orientation is associated with neural mechanisms involved in threat detection and regulation, particularly in circuits linking the amygdala, BNST, and prefrontal cortex. Yet, this association appears domain-specific and context-dependent, emerging primarily under economic or safety-related threat conditions. The absence of consistent correlations across social and economic conservatism dimensions further highlights the complexity of this link. Together, these results indicate that ideological differences in neural reactivity to threat are not fixed traits but dynamic processes influenced by the type and salience of perceived threat.

## 4. Discussion

This systematic review examines the association between political attitudes, specifically conservatism, and threat sensitivity, as well as the possible outcomes of their interaction. Of the experimental studies reviewed, ten produced physiological responses as outcomes of the association, while six were categorized as other types of responses.

Approximately three-fifths (11 of 19 studies; 57.9%) of the studies included in this review provided empirical support for a positive association between conservatism and threat sensitivity. However, a more detailed consideration of effect sizes indicates that most of these associations were of small to medium magnitude (see Supplementary Materials—Summary Tables—Tables S1–S4).

For instance, early physiological studies such as Oxley et al. [59] and Dodd et al. [63] reported modest but consistent effects (e.g., r ≈ 0.25–0.30), while subsequent replications [65,66] and cross-national analyses [67] yielded weaker or null findings. Similarly, self-reported and behavioral studies (e.g., [56,71]) tended to show small-to-moderate effects, suggesting that the link between conservatism and threat sensitivity, although recurrent, is not large in magnitude. Consistent with this, Wang et al. [74] observed small-to-medium indirect effects (ηp^2^ ≈ 0.03–0.06), showing that perceived economic threat increased xenophobia and indirectly heightened conservative attitudes through perceived group-status threat. In line with these observations, recent large-scale longitudinal evidence [75] also failed to find a consistent relationship between exposure to ecological threats and increased conservatism, reporting mixed results across national contexts.

This pattern aligns with previous work (e.g., [59]), which also found that associations between ideological orientation and threat-related responses tend to be modest. Therefore, while a majority of studies support the hypothesized positive relationship, these findings should be interpreted as reflecting a consistent but small effect, rather than a strong or universal one. The inclusion of Wang et al. [74] reinforces this conclusion by providing experimental evidence that ideological responses to threat are mediated by contextual and psychological factors rather than reflecting a fixed conservative disposition. The inclusion of large-scale longitudinal data [75] further supports the view that this association is context-dependent and may vary across cultural or temporal settings.

The presence of small effect sizes across studies also highlights the role of statistical power in shaping outcomes within this field. Several included studies were conducted with relatively small sample sizes, which may increase variability and contribute to the mixed replication results observed in the literature. By contrast, large and well-powered datasets, such as those analyzed by Cassario and Brandt [75], suggest that even when sampling issues are minimized, the association remains weak or inconsistent. Moreover, Wang et al. [74] demonstrated that even under randomized, well-controlled experimental conditions with adequate power, the magnitude of threat-related ideological shifts remains limited, further confirming the modest strength of this relationship. This is further supported by our Risk of Bias assessment (see Supplementary Materials—Tables S5–S7), which identified concerns related to sampling and confounding factors in a subset of studies. Taken together (Table 1 for a summary), these observations underscore that the relationship between political conservatism and threat sensitivity is reliable but modest, and likely moderated by contextual, cultural, and methodological factors.

The interpretation of threat sensitivity as a dynamic, context-dependent construct—rather than a fixed conservative disposition—remains supported by the available evidence, though future work should aim to improve precision through larger samples, preregistration, and consistent reporting of effect size metrics.

Overall, the findings indicate that the relationship between political conservatism and threat sensitivity is both multifaceted and context-dependent. While several non-experimental studies suggest that conservatives exhibit heightened sensitivity to a range of threats—particularly when these threats are perceived as salient to their values or group identity—experimental and physiological evidence presents a more inconsistent picture. Early research demonstrated a positive association between conservatism and physiological responses to threats, such as increased skin conductance or startle reflexes. However, subsequent replication efforts have produced mixed or null results, highlighting potential limitations in the generalizability and robustness of these effects. Importantly, more recent studies suggest that threat sensitivity among conservatives is not a fixed trait but is likely to be moderated by the type of threat (e.g., health, economic, or symbolic), ideological subdimensions (e.g., social versus economic conservatism), and sociocultural context. The lack of sub-dimension coding in our work could contribute to explaining the “mixed” results, particularly in physiological outcomes. This reinforces the need for theoretical frameworks that capture the dynamic and multidimensional nature of ideological threat responses rather than assuming a universal pattern.

The Motivated Social Cognition (MSC) model provides a central framework for understanding links between conservatism and threat sensitivity, proposing that conservative ideologies function as motivated responses to uncertainty and fear, offering cognitive closure and existential security. This account aligns with complementary perspectives: System Justification Theory (SJT) emphasizes defending existing hierarchies under threat, Terror Management Theory (TMT) highlights mortality salience as a driver of conservative adherence, and Social Dominance Theory (SDT) links outgroup threat perceptions to preferences for group-based inequality. Integrating these frameworks suggests that conservatism operates as a regulatory system for managing threat, in which heightened sensitivity functions as a protective adaptation aimed at preserving social and psychological stability.

Collectively, these frameworks help account for our findings, suggesting that conservatism reflects a multifaceted motivational response to fear and threat cues, while also explaining the variability observed across contexts, ideological subdimensions, and individual differences. Such theoretical integration transforms the interpretation of the results: rather than seeing inconsistencies as weaknesses, they can be understood as reflecting the contextual modulation of defensive motivational systems. However, it is important to note that over one-third of the literature reviewed did not confirm a robust association between conservatism and heightened threat sensitivity, with some studies reporting null or mixed effects (e.g., [66]). This pattern has been reinforced by recent cross-national evidence [75], which indicates that ideological responses to ecological threats are often weak or inconsistent across countries and time periods. This suggests that threat reactivity may not be unique to conservatism but rather connected to the intensity of ideological commitment or to the broader cognitive style of individuals positioned at the ideological extremes.

Recent perspectives argue that extremism, whether right- or left-wing, may be a stronger predictor of heightened threat perception, since extreme positions—by definition—emphasize existential urgency and rigid group boundaries [78]. In this light, large-scale studies that fail to identify systematic conservative shifts under threat conditions further support the view that threat sensitivity reflects general ideological rigidity rather than conservatism per se. This insight broadens the conceptual scope of the discussion by proposing that threat sensitivity reflects ideological rigidity in general, rather than conservatism alone, offering a bridge between political psychology and the study of cognitive-motivational processes underlying extremism.

Regarding neural responses, fMRI evidence indicates that conservatism is associated with activation in regions such as the amygdala which are directly involved in threat perception and regulation, while liberalism shows stronger activation in ACC, a region involved in conflict monitoring [79,80]. However, Nash and Leota [77], reported heightened ACC activation for conservatism in threat conditions as reflected in P3 responses, suggesting that the relationship between conservatism and ACC activity is context-dependent, particularly in situations involving threat.

Moreover, Yang et al. [81] reported that economic conservatism predicts stronger activation in BNST, as well as enhanced connectivity with the amygdala which is in line with high activation found under threat condition by Pederson et al. [80]. Similarly, threat-elicited responses in the amygdala and BNST have been positively correlated with threat-induced skin conductance responses (SCR) [82,83,84]. Together, these results suggest a neural network mechanism that may underlie heightened physiological reactivity to threat. These convergences between neural, physiological, and attitudinal data reinforce the idea that ideological threat sensitivity operates through an integrated neurocognitive system that regulates responses to uncertainty and danger.

## 5. Conclusions and Future Directions

In this systematic review, key findings from various studies were examined to elucidate the correlation between conservatism and threat sensitivity. Overall, while the majority of studies support the notion that conservatives tend to exhibit heightened sensitivity to negative or threatening stimuli, the strength of this association is generally small to moderate in magnitude (see Supplementary Materials—Summary Tables). This observation is consistent with prior evidence (e.g., [59]), suggesting that the conservatism–threat sensitivity relationship, while statistically reliable in many cases, represents a modest effect rather than a large or universal one. The inclusion of Wang et al. [74], the only randomized controlled trial identified in this review, further supports this conclusion: despite employing an experimental manipulation of economic threat under well-controlled conditions, the observed effects were small to medium in size. These findings reinforce the view that ideological shifts in response to threat are limited in magnitude and mediated by psychological factors such as perceived group-status threat rather than reflecting fixed dispositional tendencies.

Recent large-scale longitudinal evidence [75] further supports this interpretation, indicating that the association between threat exposure and conservatism is often weak, inconsistent, and strongly dependent on cultural and temporal context. Together, these results highlight that both experimental and correlational evidence converge toward the same conclusion: the relationship between conservatism and threat sensitivity is contextually contingent and modest in strength, even under randomized or ecologically valid conditions. Moreover, the limited statistical power and small sample sizes in several of the included studies may have contributed to variability and replication challenges observed in this literature. Theoretically, this synthesis advances the understanding of political ideology as a motivational and regulatory system. By integrating perspectives from the MSC model, SJT, TMT, and SDT, we conceptualize conservatism not as a fixed trait but as a flexible and context-dependent adaptation to perceived threat, uncertainty, and loss of control. Within this framework, threat sensitivity can be understood as a protective mechanism that promotes psychological stability and social order under conditions of perceived danger. However, recent experimental findings (e.g., [74]) suggest that such adaptive responses may also emerge among individuals across the ideological spectrum when the perceived threat is personally relevant, emphasizing the dynamic and reciprocal nature of threat–ideology interactions.

A limitation concerns the inconsistency in effect size reporting across the included studies, which constrained our ability to perform quantitative synthesis. Future research should address this by systematically reporting standardized effect size metrics and ensuring adequate statistical power through larger, preregistered samples. Additionally, expanding database coverage (e.g., PsycINFO, Web of Science, Scopus) and employing AI-assisted screening tools will enhance precision and reproducibility.

Future research should not only aim to replicate existing findings but also refine theoretical frameworks by systematically examining moderators such as cultural context, ideological subdimensions, and interoceptive sensitivity. Multimethod approaches integrating physiological, neural, and self-report measures will be essential for building a comprehensive model of threat sensitivity as a multilevel construct encompassing emotional, cognitive, and sociocultural dimensions. Incorporating paradigms such as Pavlovian fear conditioning [40,85,86] and interoceptive awareness measures [87,88,89] may further clarify the mechanisms linking threat processing and ideological orientation.

## Figures and Tables

**Table 1:** Summary of observations.

Study	Measurement Modalities	Support For Link	Notes
Oxley et al. (2008) [59]	SCL	Yes	Increased skin conductance linked to social conservatism
Dodd et al. (2012) [63]	SCL, Eye-tracking	Yes	Greater physiological and attentional responses to threat among conservatives
Arceneaux et al. (2018) [64]	SCL	Yes	Higher physiological arousal linked to conservative policy preferences
Knoll et al. (2015) [65]	SCL	No	Failed to replicate physiological link between conservatism and threat
Bakker et al. (2020) [66]	SCL	No	No significant link found in three replication studies
Osmundsen et al. (2022) [67]	EDA	No	No significant association in Danish or U.S. samples via EDA
Arceneaux et al. (2024) [68]	SCL	No	No significant association between threat and conservatism
Eadeh and Chang (2020) [69]	Self-reported	No	Threat sensitivity is more correlated with liberal ideology
Nowlan and Zane (2020) [55]	Self-reported	Yes	Conservatives adjust behavior under high perceived threat
Brandt et al. (2021) [70]	Self-reported	Yes	Significant association between right-wing beliefs and perceived threat
Pazhoohi and Kingstone (2021) [71]	Self-reported	Yes	Increased disease threat linked to higher conservatism
Lukaszewicz (2022) [72]	Self-reported	Partial	Only social and securitarian conservatives showed heightened threat perception
Osmundsen et al. (2022) [67]	Self-reported	Partial	Self-reported negativity correlated with social conservatism only
Brown et al. (2024) [73]	Self-reported	Yes	The correlation between parasite stress and conservatism was stronger for older participants
Syfers et al. (2023) [56]	Self-reported	Yes	Conservatives report higher symbolic and realistic threat, esp. under uncertainty
Pedersen et al. (2018) [50]	Neuroimaging (fMRI)	No	Economic, not social, conservatism linked to amygdala-BNST connectivity; limited generalizability
Nash and Leota (2022) [77]	Neurocognitive (EEG)	Yes	Conservatives show greater P3 response under economic threat
Wang et al. (2024) [74]	Self-Reported	Yes	Economic threat indirectly heightened conservatism and racial bias through group-status threat.
Cassario & Brandt (2024) [75]	Self-reported	No	No consistent conservative shift
Mills et al. (2016) [76]	Self-reported	Yes	Conservatives show better memory for threat-related stimuli

Note: Studies were categorized as “Yes” if they provided clear empirical support, “Partial” if support was conditional (e.g., on subgroups), and “No” if they failed to support or replicate the hypothesized relationship.

## Data Availability

No new data were created or analyzed in this study.

## Supplementary Materials

The following supporting information can be downloaded at: https://www.mdpi.com/article/10.3390/brainsci15111191/s1, Table S1: Summary of Quantitative Studies; Table S2: Summary of Physiological Studies; Table S3: Summary of Failed Reproduciton of Physiological Studies; Table S4: Summary of Studies with other responses as outcome; Table S5: JBI Critical Appraisal for Quantitative Studies; Table S6: Risk of Bias Assessment for Non-randomized Trial Studies Using ROBINS-I V2 Assessment Tool; Table S7: Risk of Bias Assessment for Randomized Trial Studies Using RoB 2 Assessment Tool.
